# Free-breathing 3D Stack-of-Stars Gradient Echo Sequence in MR-guided Percutaneous Liver Interventions: Evaluation of Workflow and Diagnostic Quality

**DOI:** 10.1007/s00270-022-03350-5

**Published:** 2023-01-06

**Authors:** Julian Glandorf, Dominik Horstmann, Daniel Markus Düx, Frank Wacker, Marcel Gutberlet, Bennet Hensen

**Affiliations:** Institute for Diagnostic and Interventional Radiology, Hannover Medical School, Carl-Neuberg-Str. 1, 30625 Hannover, Germany

**Keywords:** MR-guidance, IMRI, Percutaneous liver interventions, StarVIBE, Stack-of-stars, Free-breathing imaging

## Abstract

**Purpose:**

To evaluate workflow efficiency and diagnostic quality of a free-breathing 3D stack-of-stars gradient echo (Radial GRE) sequence compared to a breath-hold 3D Cartesian gradient echo (Cartesian GRE) sequence for needle position control in MR-guided liver interventions.

**Materials and Methods:**

12 MR-guided liver interventions were performed on a 1.5 T Siemens Aera and analyzed retrospectively. 15 series of the Radial GRE sequence were compared to 14 series of the Cartesian GRE sequence regarding the time interval between two consecutive live-scans for needle tracking (Tracking-2-Tracking-Time). The quality of both sequences was compared by the SNR within comparable slices in liver and tumor ROIs. The CNR was calculated by subtraction of the SNR values. Subjective image quality scores of three radiologists were assessed and inter-rater reliability was tested by Fleiss’ kappa. Values are given as mean ± SD. *P*-values < 0.05 were considered as significant.

**Results:**

The median Tracking-2-Tracking-Time was significantly shorter for the Radial GRE sequence, 185 ± 42 s vs. 212 ± 142 s (*p* = 0.04) and the median SNR of the liver and tumor ROIs were significantly higher in the Radial GRE sequence, 249 ± 92 vs. 109 ± 67 (*p* = 0.03) and 165 ± 74 vs. 77 ± 43 (*p* = 0.02). CNR between tumor and liver ROIs showed a tendency to be higher for the Radial GRE sequence without significance, 68 ± 48 vs. 49 ± 32 (*p* = 0.28). Mean subjective image quality was 3.33 ± 1.08 vs. 2.62 ± 0.95 comparing Radial and Cartesian GRE with a Fleiss’ kappa of 0.39 representing fair inter-rater reliability.

**Conclusion:**

A free-breathing 3D stack-of-stars gradient echo sequence can simplify the workflow and reduce intervention time, while providing superior image quality. Under local anesthesia, it increases patient comfort and reduces potential risks for needle dislocations in MR-guided liver interventions by avoiding respiratory arrests for needle position control.

**Supplementary Information:**

The online version contains supplementary material available at 10.1007/s00270-022-03350-5.

## Introduction

MR-guidance for interventional procedures is exceptional by its combination of morphological display with functional information allowing extraordinary visibility and immediate therapy control [1, 2]. However, it has to overcome its technical challenges to play a larger role among the other modalities.

Most sequences use a Cartesian image acquisition scheme, in which the k-space is sampled across parallel lines with a fixed phase offset, which causes phase distortions and ‘ghost artifacts’ in moving structures [3]. This is a mayor challenge for MRI-guidance within the moving organs due to the need for accuracy and sufficient image quality [4].

This challenge might be overcome by a recently implemented free-breathing 3D stack-of-stars gradient echo sequence (Radial GRE), in which the data are acquired along rotating radial spokes through the center of k-space resulting in a star-shaped readout in the k_x_- and k_y_-plane. A cylindrical volume with a ‘stack-of-stars’ is generated by a conventional sampling in the k_z_-dimension. The repetitive readout of the center of k-space averages phase shifts of the low frequency components caused by motion [5]. This enables the unique ability to generate a 3D dataset during shallow breathing and creates higher patient comfort, less motion artifacts and a simplified workflow for interventions by avoiding breath-holds.

Challenges of the Radial GRE are potentially longer scan durations, sophisticated image reconstruction, necessity of high magnetic field homogeneity and precise time-varying gradient fields [3]. Fortunately, most of the technical burdens have been solved during the recent decades allowing a more widely implementation of radially acquired sequences [5].

Therefore, the purpose of this study is to evaluate the workflow efficiency and the diagnostic quality of a Radial GRE sequence compared to a conventional breath-hold 3D Cartesian gradient echo (Cartesian GRE) sequence for needle position control in MR-guided liver interventions.

## Materials and Methods

### Data Acquisition

Percutaneous liver procedures were performed on a 1.5 T MRI (*MAGNETOM Aera*, Siemens Healthineers, Erlangen, Germany) using a 4-channel flex coil and the spine coil (24 channels) for lesions showing weak or no visibility in computed tomography and sonography (Supporting Information Fig. S1). Only one lesion (hepatocellular carcinoma) was hyperintense on the T1-weighted sequence. All other lesion were hypointense on the T1-weighted sequence and predominantly hyperintense on the T2-weighted sequence. Thirteen interventions were analyzed retrospectively. One dataset was performed during a clinical training and was excluded. The included patient cohort consisted of 11 patients (one patient had two biopsies) with metastases of colon cancer (1), medullary thyroid cancer (patient with two biopsies), sarcomas (2) and seven patients with hepatocellular carcinomas. Most interventions (8/12) were microwave liver ablations performed under general anesthesia and one third (4/12) were MR-guided biopsies performed under local anesthesia.

**Fig. 1:**
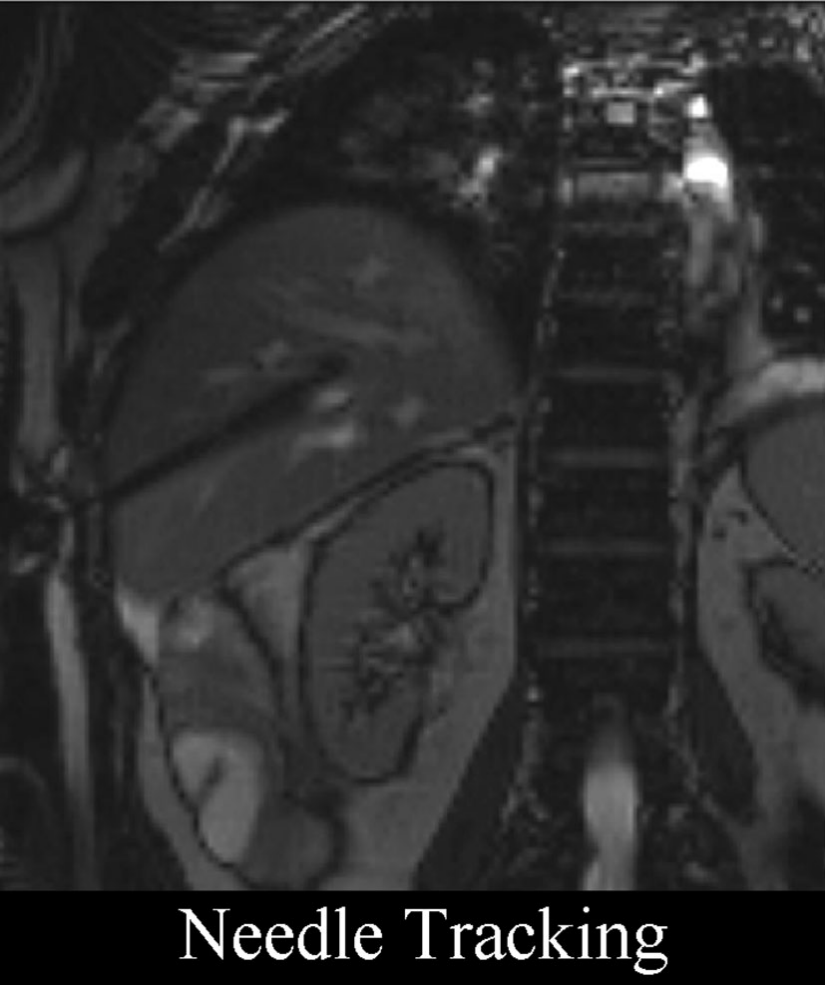
Exemplary image of an interactive real-time coronal balanced steady-state free precession sequence for needle tracking

An interactive real-time balanced steady-state free precession sequence (*BEAT IRTTT*, Siemens) with three parallel slices was used for needle tracking (Fig. 1). The needle tracking sequence is a 2D sequence to follow the needle placement in real-time, which increases safety of the intervention and the confidence of the interventionalist. However, due to its relatively low resolution and only 2D visualization, the exact location of the very thin needle tip is not visible (Supporting Information Fig. S2). Therefore, 3D datasets are necessary to confirm the needle position. For this, a free-breathing 3D stack-of-stars gradient echo sequence (*StarVIBE*, Siemens) or a breath-hold 3D Cartesian gradient echo sequence (*VIBE*, Siemens) were used repetitively until the final needle position was reached. Free-breathing and breath-hold for patients under general anesthesia is considered as the respirator being turned on or paused. Protocol parameters are depicted in Table 1.

**Table 1:** Sequence parameters of the radially acquired free-breathing 3D stack-of-stars gradient echo sequence (Radial GRE) and the breath-hold 3D Cartesian gradient echo sequence (Cartesian GRE). Despite heterogeneity, the resolution of the Radial GRE sequence was higher in all cases. Repetition time (TR), echo time (TE)

Sequence parameter	Radial GRE sequence	Cartesian GRE sequence
TR [ms]	4	3.34
TE [ms]	1.55	1.19
Flip angle [°]	12	12
Pixel bandwidth [Hz/pixel]	601	476
Resolution [mm]	1–1.41^2^ Mean: 1.33 SD: 0.14	1.41–1.55^2^ Mean: 1.49 SD: 0.43
Slice thickness [mm]	2–4 Mean: 2.5 SD: 0.71	2–3 Mean: 2.25 SD: 0.43
Scan time [s]	52	17

### Workflow and Image Analysis

Radial or Cartesian GRE were used to plan the trajectories of the intervention. T2-weighted or a post-contrast T1-weighted sequence are only used, if the lesion is not visible otherwise. Then, finger tipping is performed during the tracking sequence to validate and mark the entry point on the surface of the patient. Then, the needle tracking sequence is also used during needle placement. The position is controlled repetitively either by the Radial or Cartesian GRE. The ablation zone is controlled by T1-weighted post-contrast series and a T2-weighted sequence with fat saturation.

**Fig. 2:**
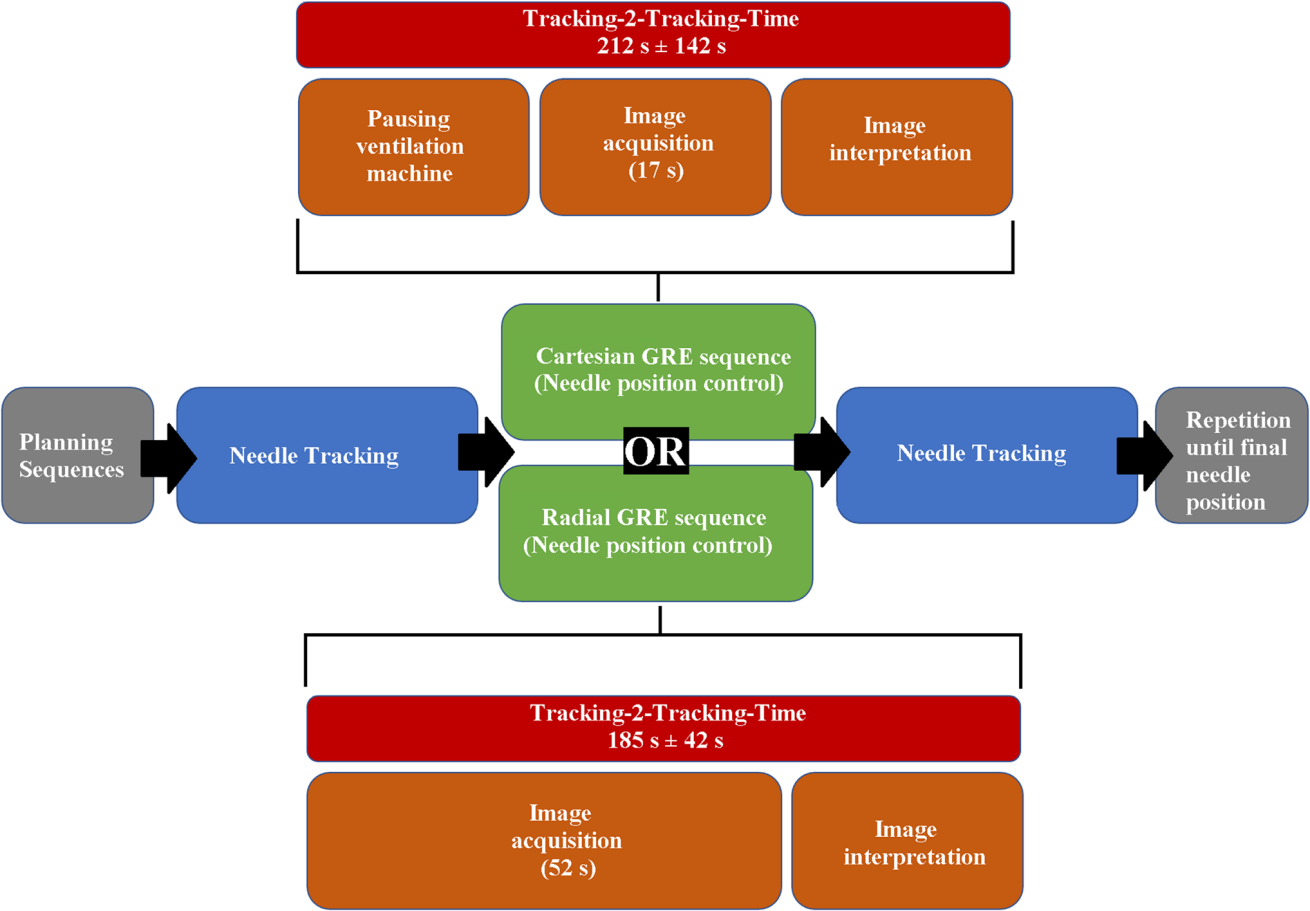
Schematic workflows of the percutaneous MR-liver interventions. Planning and needle position control is performed using the 3D datasets of the breath-hold 3D Cartesian gradient echo sequence (Cartesian GRE) or the radially acquired free-breathing 3D stack-of-stars gradient echo sequence (Radial GRE)

In total, 15 series of the Radial GRE sequence from a cohort of five patients were compared to 14 series of the Cartesian GRE sequence of a cohort of six patients in terms of their workflow efficiency, which was evaluated by the time interval between two consecutive live-scans for needle tracking (Tracking-2-Tracking-Time) (Fig. 2).

The SNR comparison was performed on eight slices showing tumor and healthy liver from the aforementioned cohort of five patients that had both Radial with corresponding Cartesian GRE datasets for planning.

**Fig. 3:**
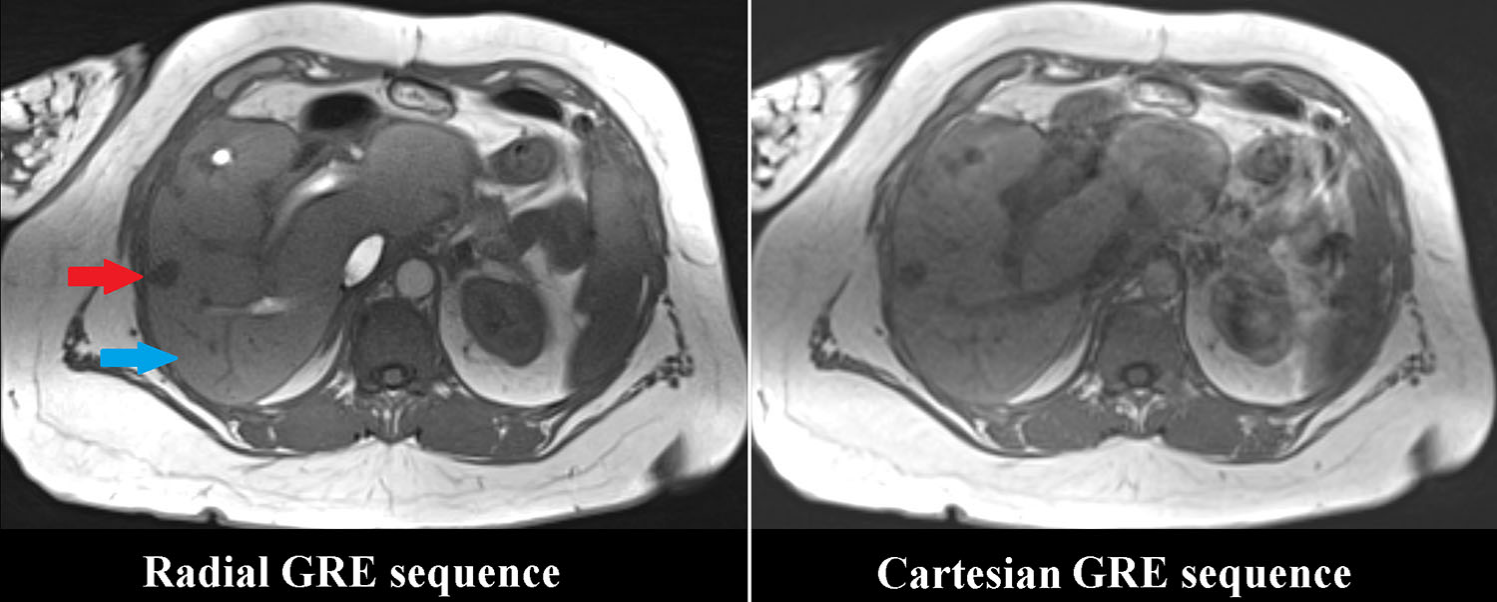
Example of two matching slices of the radially acquired free-breathing 3D stack-of-stars gradient echo sequence (Radial GRE) and the breath-hold 3D Cartesian gradient echo sequence (Cartesian GRE) sequence. The SNR was compared between identical ROIs within the tumor (red arrow) and healthy liver (blue arrow). Stronger flow-related enhancement within venous vessels is visible in the Radial GRE sequence

The quality of both sequences was compared on matched slices within identical ROIs of the tumor and liver. Considering the non-central chi distribution in magnitude images of multichannel receive coils, the signal-to-noise-ratio (SNR) was estimated from the ratio of the mean signal in a ROI within the liver parenchyma or tumor and the standard deviation of the background signal (SD) (Fig. 3) [6]. The contrast-to-noise-ratio (CNR) was calculated by (SNR_Liver_-SNR_Tumor_)/SD. The subjective image quality of both sequences was rated visually by three interventional radiologists using a score from 1 (very low quality) to 5 (very high quality). The inter-rater reliability was calculated using Fleiss’ kappa.

The statistical analyses were performed using MATLAB (The MathWorks, Natick, MA). Since normal distribution was not given in the Shapiro–Wilk Test, the nonparametric Mann–Whitney U Test was used to compare the central tendencies. The values are given in median ± standard deviation. P-values < 0.05 were considered as significant.

## Results

Technical success was observed in all procedures and no major complications like acute bleeding, pneumothorax, bowel perforation or infection occurred. One patient had a re-biopsy due to poor histological quality.

The median age of the participants was 69 ± 10 years and consisted of four women and seven men. In total, 17 lesions were treated in 12 interventions resulting in 1.42 ± 1.11 lesions per intervention. The average number of breathing stops (considering the use of a Cartesian GRE) was 12.08 ± 5.35 per intervention. The average lesion size was 13.95 ± 9.32 mm.

The median Tracking-2-Tracking-Time was significantly shorter for the Radial compared to the Cartesian GRE sequence, 185 ± 42 s vs. 212 ± 142 s (*p* = 0.04). Considering 12.08 breathing stops per intervention, the use of a Radial GRE reduces total median intervention time not significantly from 125 ± 59 min to 119 ± 59 min by 5:26 min (*p* = 0.69).

In addition, the median SNR of the liver ROIs was significantly higher in the Radial GRE sequence in comparison to the Cartesian GRE sequence, 249 ± 92 vs. 109 ± 67 (*p* = 0.03). Also, the median SNR of the lesion ROIs was significantly higher in the Radial compared to the Cartesian GRE sequence, 165 ± 74 vs. 77 ± 43 (*p* = 0.02).

Without significance, the CNR between the tumor ROIs and the liver ROIs showed a tendency to be higher for the Radial GRE sequence with median values of 68 ± 48 vs. 49 ± 32 in the Cartesian GRE sequence (*p* = 0.28).

The mean subjective image quality score for the Radial GRE was 3.33 ± 1.08 vs. 2.62 ± 0.95 for the Cartesian GRE sequence. Fleiss’ kappa was 0.39 leading to a fair inter-rater agreement regarding Landis and Koch [7].

**Fig. 4:**
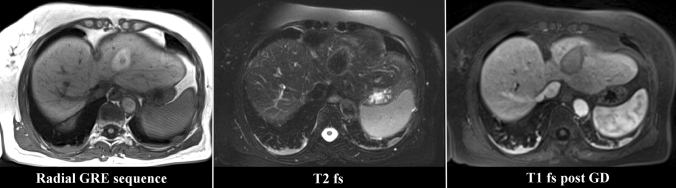
Visibility of the ablation zone indicated by parenchymal hemorrhage in the radially acquired free-breathing 3D stack-of-stars gradient echo sequence (Radial GRE). T2-weighted fat saturated (T2 fs) and T1-weighted fat saturated after gadolinium-based contrast (T1 fs post GD) are shown for comparison

Also, the Radial GRE sequence presented good visualization of the parenchymal hemorrhage in the center of an ablation zone after therapy (Fig. 4).

## Discussion

The interventional workflow in a diagnostic MR environment is often cumbersome and requires workarounds. This increases complexity, intervention time and healthcare costs [8–12]. Any reduction of intervention time by the avoidance of breath-holds can offer an advantage in this regard.

Our results indicate benefits of the Radial GRE sequence in the setting of MR-guided liver interventions in terms of workflow improvements, safety considerations and diagnostic quality.

**Fig. 5:**
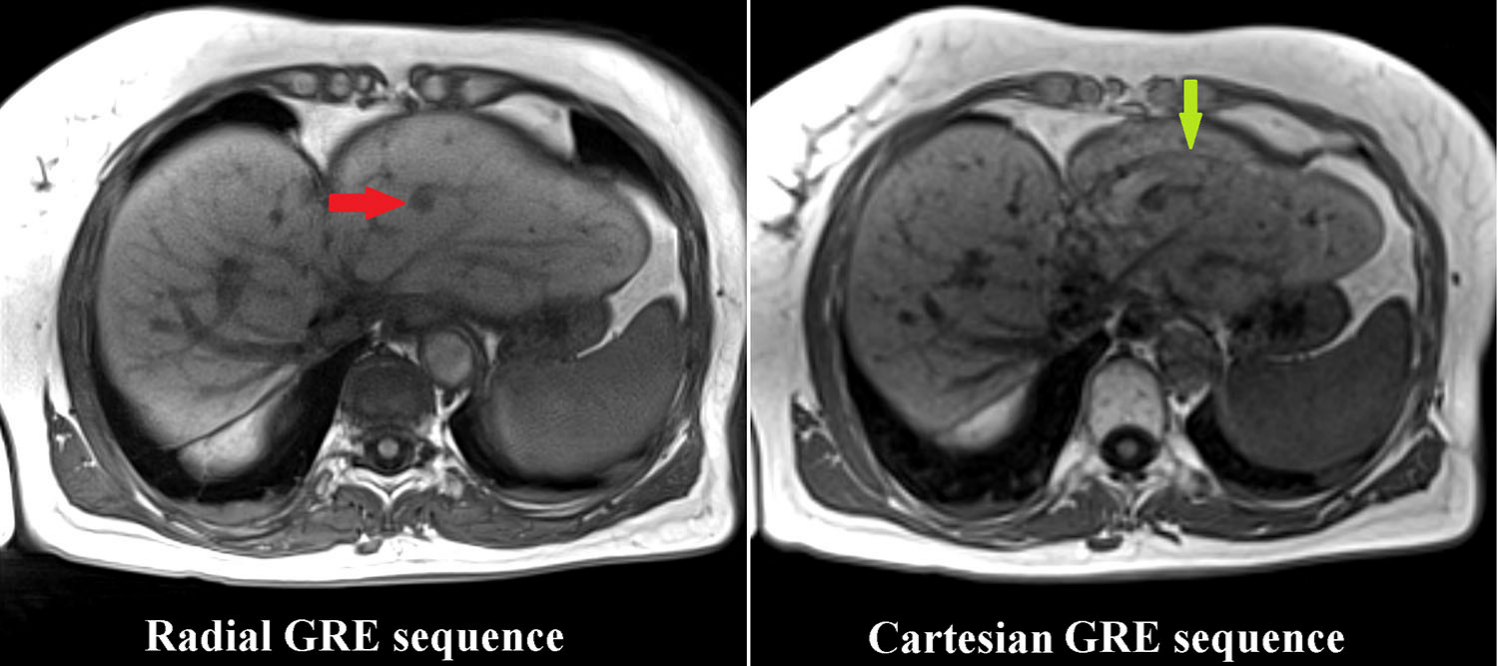
Comparison of two matching slices of the radially acquired free-breathing 3D stack-of-stars gradient echo sequence (Radial GRE) and the breath-hold 3D Cartesian gradient echo sequence (Cartesian GRE). The tumor (red arrow) is visible in both sequences. However, the Cartesian GRE sequence depicts a ghost artifact of the moving heart (green arrow)

Beside superior SNR, which is in-line with previous reports [13], the Radial GRE sequence inherits the advantage of being sampled radially being more robust against motion artifacts (Fig. 5) [5, 14]. Another benefit using the Radial GRE is the increase of comfort for patients being treated under local anesthesia by avoiding painful breath-holds during needle placement. Furthermore, the risk of a needle dislocation decreases without extreme movement of the diaphragm, which might be needed for longer breath-holds.

Due to the limited number of patients, our study has the limitation of a relatively heterogeneous group of procedures. Nevertheless, the workflows of MR-guided biopsies and MR-guided ablations are very similar. Since the Tracking-2-Tracking-Time is a compound of pausing the ventilation machine (only for Cartesian GRE sequence, which depends on anesthesia team), scan duration (52 s for the Radial GRE sequence compared to 17 s for the Cartesian GRE sequence) and image interpretation (difficulty of the case, experience of the interventionalist), there are many variables that influence procedure time. As the scan time of the Radial GRE sequence is longer than the Cartesian GRE sequence, time is saved by avoiding the pausing of the ventilation machine and, maybe, during image interpretation, given the higher SNR. The very high standard deviation of the Cartesian GRE sequence group indicates, that the intervention times is quite unpredictable.

Another limitation of our study is the minimal variation of the image resolution within each group. The number of slices and spokes of the Radial GRE sequence was selected to shorten scan time while maintaining image quality and practicability during interventions. However, the resolution of the Radial GRE sequence was higher for all patients allowing good visualization also in reformatted images (Supporting Information Fig. S2). When applying the same resolution as for the Cartesian GRE sequence, a further increase of the SNR might be possible. Furthermore, less motion artifacts in patients under general anesthesia are likely. However, the number of procedures was too little for this subgroup analysis.

## Conclusion

The free-breathing 3D stack-of-stars gradient echo sequence can simplify the workflow, reduce intervention time, increase patient comfort and decrease potential risk for needle dislocations in MR-guided liver interventions by avoiding respiratory arrests, while providing excellent image quality.

## Supplementary Information

Below is the link to the electronic supplementary material.

Supplementary file1 (DOCX 2755 KB)
