# Supplementary material for: Free-breathing 3D Stack-of-Stars Gradient Echo Sequence in MR-guided Percutaneous Liver Interventions: Evaluation of Workflow and Diagnostic Quality
Source: Cardiovasc Intervent Radiol. 2023 Jan 6;46(2):274–9. doi: 10.1007/s00270-022-03350-5 (PMC9892107; doi:10.1007/s00270-022-03350-5)
Supplement: Supplementary file 1 — Supplementary file1 (DOCX 2755 KB) [file 270_2022_3350_MOESM1_ESM.docx]

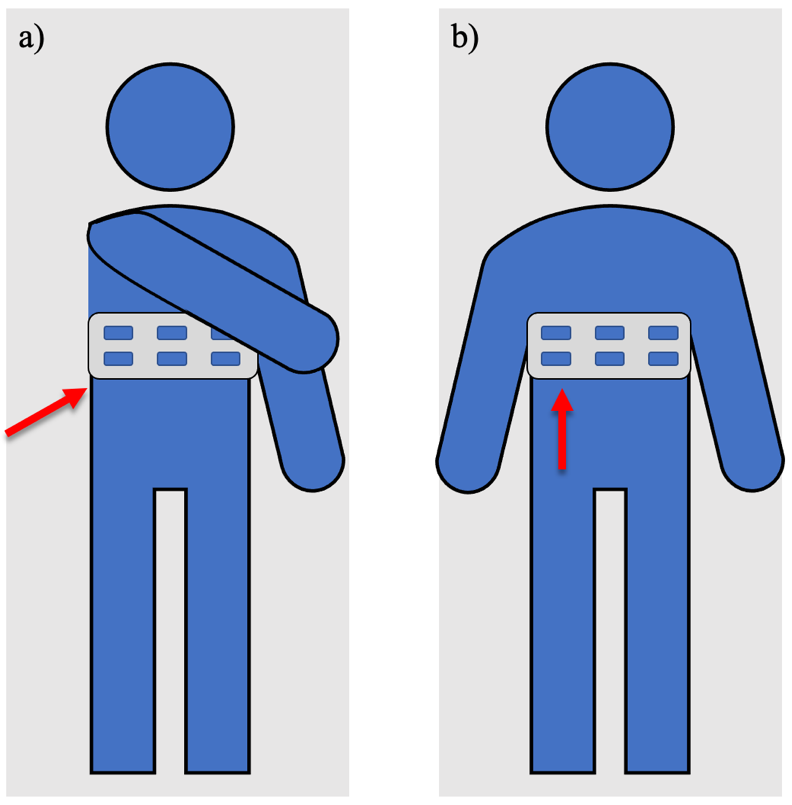


**Supporting Information Fig. S1** Schematic illustration of possible flex coil positions on the upper abdomen for percutaneous liver interventions. In a), the patient’s arm is placed to the side enabling an intercostal trajectory from the right patient side within a coronal plane. In b), a subdiaphragmal approach is indicated entering directly below the flex coil allowing a trajectory within a sagittal plane. Notably, combinations with more difficult trajectories using double angulations are possible


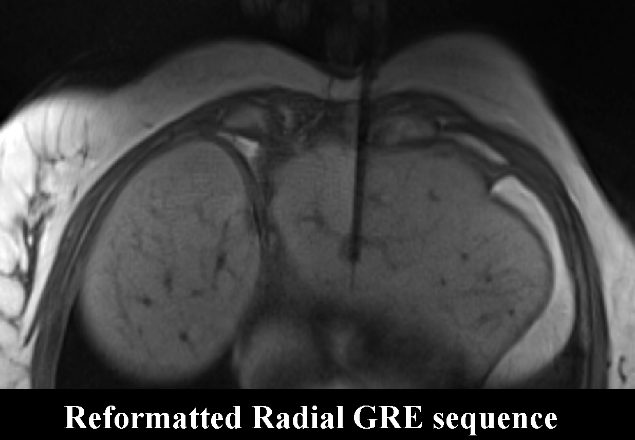


**Supporting Information Fig. S2** Position control after needle placement in the tumor. Almost isotropic resolution of the radially acquired free-breathing 3D stack-of-stars gradient echo sequence (Radial GRE) allows good image quality in reformatted image planes (reformation of the Radial GRE sequence shown in Fig. 4). Notice the visibility of the very thin active needle tip of the ECO needle system (ECO Medical Instruments, Nanjing, Jiangsu, China)
